# Preoperative Chemoradiotherapy Versus Perioperative Chemotherapy for Patients With Resectable Esophageal or Gastroesophageal Junction Adenocarcinoma

**DOI:** 10.1245/s10434-017-5827-1

**Published:** 2017-04-19

**Authors:** M. C. J. Anderegg, P. C. van der Sluis, J. P. Ruurda, S. S. Gisbertz, M. C. C. M. Hulshof, M. van Vulpen, N. Haj Mohammed, H. W. M. van Laarhoven, M. J. Wiezer, M. Los, M. I. van Berge Henegouwen, R. van Hillegersberg

**Affiliations:** 1 Department of Surgery, Academic Medical Center, Amsterdam, The Netherlands; 2 Department of Surgery, University Medical Center Utrecht, Utrecht, The Netherlands; 3 Department of Radiation Oncology, Academic Medical Center, Amsterdam, The Netherlands; 4 Department of Radiation Oncology, University Medical Center Utrecht, Utrecht, The Netherlands; 5 Department of Clinical Oncology, University Medical Center Utrecht, Utrecht, The Netherlands; 6 Department of Clinical Oncology, Academic Medical Center, Amsterdam, The Netherlands; 7 Department of Surgery, Antonius Hospital, Nieuwegein, The Netherlands; 8 Department of Internal Medicine and Oncology, Antonius Hospital, Nieuwegein, The Netherlands

## Abstract

**Background:**

This study compares neoadjuvant chemoradiotherapy (nCRT) with perioperative chemotherapy (pCT) for patients with resectable esophageal or gastroesophageal junction (GEJ) adenocarcinoma in terms of toxicity, postoperative complications, pathologic response, and survival.

**Methods:**

This study retrospectively analyzed and compared 313 patients with resectable esophageal or GEJ adenocarcinoma treated with either nCRT (carboplatin/paclitaxel 41.4 Gy, *n* = 176) or pCT (epirubicin, cisplatin and capecitabine, *n* = 137).

**Results:**

The baseline and tumor characteristics were similar in both groups. The ability to deliver all planned preoperative cycles was greater in the nCRT group (92.0 vs. 76.6%). Whereas nCRT was associated with a higher rate of grades 3 and 4 esophagitis, pCT was associated with a higher rate of grades 3 and 4 thromboembolic events, febrile neutropenia, nausea, vomiting, diarrhea, hand–foot syndrome, mucositis, cardiac complications, and electrolyte imbalances. Two patients in the pCT group died during neoadjuvant treatment due to febrile neutropenia. More postoperative cardiac complications occurred in the nCRT group. All other postoperative complications and the in-hospital mortality rate (nCRT, 4.7%; pCT, 2.3%) were comparable. The pathologic complete response (pCR) rate was 15.1% after nCRT and 6.9% after pCT. Radicality of surgery was comparable (R0: 93.0 vs. 91.6%). The median overall survival was 35 months after nCRT versus 36 months after pCT.

**Conclusion:**

For patients with esophageal or GEJ adenocarcinoma, chemoradiotherapy with paclitaxel, carboplatin and concurrent radiotherapy, and perioperative chemotherapy with epirubicin, cisplatin, and capecitabin lead to equal oncologic outcomes in terms of radical resection rates, lymphadenectomy, patterns of recurrent disease, and (disease-free) survival. However, neoadjuvant chemoradiotherapy is associated with a considerably lower level of severe adverse events and should therefore be the preferred protocol until a well-powered randomized controlled trial provides different insights.

**Electronic supplementary material:**

The online version of this article (doi:10.1245/s10434-017-5827-1) contains supplementary material, which is available to authorized users.

Esophageal and gastroesophageal junction (GEJ) adenocarcinomas usually are diagnosed in an advanced stage.^1^ Due to rapid dissemination, the prognosis is dismal for the majority of patients, resulting in overall survival rates of 15–25%.^1^^,^ 2 Surgical resection is the cornerstone of curative treatment for selected patients without distant metastases. The key objective for this surgical approach is to achieve a radical (R0) resection with an appropriate lymphadenectomy.

Unsatisfactory results of surgery without neoadjuvant therapy incited development of multimodal approaches in the treatment of esophageal cancer.^3^ Multiple randomized clinical trials have shown that both neoadjuvant chemoradiotherapy (nCRT) and perioperative chemotherapy (pCT) confer a survival benefit compared with surgery alone.^4^–^9^ The results of an updated meta-analysis are in concordance with these results and provide strong evidence for a survival benefit of neoadjuvant chemoradiotherapy or chemotherapy over surgery alone for patients with esophageal adenocarcinoma.^9^

To date, direct evidence comparing nCRT and pCT has been limited to three small randomized controlled trials and has been inconclusive regarding patient outcomes such as postoperative morbidity, mortality, radicality of surgery, and survival.^9^–^12^

In Europe and North America, chemoradiotherapy currently is the preferred neoadjuvant strategy. The most widely used chemoradiation regimen, with paclitaxel, carboplatin, and 41.4 Gy/23 fractions radiotherapy, is based on the CROSS-2 trial.^8^

In the United Kingdom, perioperative chemotherapy with epirubicin, cisplatin, and fluorouracil (ECF) is considered the standard of care, based on the OEO 2 study and the MAGIC trial.^4^^,^ 5 After publication of the REAL2 trial, in which the noninferiority of substituting oral capecitabine for infused 5-fluorouracil (5-FU) was shown, ECF was changed to ECX (epirubicin, cisplatin, and capecitabine) in many clinics.^13^^,^ 14 To date, no direct comparisons have been made between pCT with ECX chemotherapy and nCRT with paclitaxel, carboplatin, and 41.4 Gy/23 fractions.

This study aimed to compare perioperative ECX-based chemotherapy and chemoradiotherapy with paclitaxel, carboplatin, and concurrent radiotherapy in terms of toxicity, postoperative complications, pathologic response, long-term survival, and disease recurrence.

## Patients and Methods

### Patient Population

Between April 2005 and November 2011, patients with resectable esophageal or junctional adenocarcinoma were treated at three high-volume referral centers in The Netherlands with two different neoadjuvant regimens. At the Academic Medical Center (Amsterdam, The Netherlands), patients received neoadjuvant chemoradiotherapy (nCRT). At the University Medical Center Utrecht (Utrecht, The Netherlands) and Antonius Hospital (Nieuwegein, the Netherlands), patients received perioperative chemotherapy (pCT).

All patients who started neoadjuvant treatment were included in the analysis. They had World Health Organization (WHO) performance statuses of 0–2. Underlying diseases such as cardiac, vascular, pulmonary, or oncologic (other than esophageal) disorders had to be stable and under the control of their treating physician. All patients were discussed in a multidisciplinary oncology meeting of surgeons, gastroenterologists, medical oncologists, radiation oncologists, and radiologists before treatment. The patients were not asked to provide informed consent for this specific study because the data were primarily recorded as part of standard care. The local ethics committees approved this approach.

### Data Collection

The primary end points of the study were toxicity, postoperative complications, pathologic response, long-term survival, and disease recurrence. Data were extracted from the prospectively collected databases of all the centers. The baseline characteristics included age, sex, body mass index (BMI), comorbidity, and American Society of Anesthesiology (ASA) score. The routine diagnostic workup included upper endoscopy with biopsy, endoscopic ultrasound (EUS), computed tomography (CT) of the thorax and abdomen, and ultrasound of the neck region. Integrated^18^F-fluorodeoxyglucose positron emission tomography (FDG-PET/)CT scanning and fine-needle aspiration (FNA) of suspected lymph nodes were used when indicated.

Pre- and postoperative treatment characteristics were collected, including chemotherapy regimens, number of chemotherapy doses, dose reductions, dose density, and the necessity to interrupt or cease treatment because of adverse events.

### Chemotherapy

The patients were scheduled to receive three preoperative ECX chemotherapy cycles and three postoperative ECX cycles. The pre- and postoperative chemotherapy cycles comprised intravenous administration of epirubicin (50 mg/m^2^) and cisplatin (60 mg/m^2^), followed by 1000 mg/m^2^ of capecitabine twice daily for 14 days or 625 mg/m^2^ of capecitabine twice daily for 21 days. Adaptations to the regimen such as dose reduction or change of regimen to oxaliplatin or 5-FU were applied when necessary. A second CT scan after the second course of chemotherapy was performed to monitor the therapeutic effect.

### Chemoradiotherapy

On days 1, 8, 15, 22, and 29, carboplatin at 2 mg/mL per min targeted to an area under the curve and paclitaxel at a dose of 50 mg per square meter of body surface area were administered intravenously. A total radiation dose of 41.4 Gy conformal external beam radiotherapy was administered in 23 fractions of 1.8 Gy each, with five daily fractions per week, starting on the first day of the first chemotherapy cycle.^8^

### Toxicity

Source data verification of all grade 3 and higher adverse events was performed by two separate observers (P.C.S., M.C.J.A.) according to the National Cancer Institute Common Terminology Criteria for Adverse Events, version 4.0.11. Grades 3, 4, and 5 adverse events were graded by consensus of two authors (P.C.S., M.C.J.A.).

### Surgery

Different types of open and minimally invasive transthoracic and transhiatal surgery were performed during the inclusion period. Esophagectomy was performed by means of a transthoracic or transhiatal approach. A three-stage (minimally invasive) transthoracic esophagectomy was the standard surgical approach. Patients with a tumor located at the GEJ or with reduced performance status (and therefore inability to undergo transthoracic esophagectomy) underwent transhiatal esophagectomy.

In short, during transhiatal esophagectomy, the esophagus was dissected under direct vision through the widened hiatus of the diaphragm up to the inferior pulmonary vein. The tumor and its adjacent lymph nodes were dissected en bloc. The paracardial, lesser curvature, left gastric artery (together with the lesser curvature), celiac trunk, common hepatic artery, and splenic-artery nodes were dissected, and a 3 cm-wide gastric tube was constructed. After left-sided mobilization of the cervical esophagus, the intrathoracic esophagus was bluntly stripped from the neck to the upper level of the inferior pulmonary vein using a vein stripper.

The transthoracic esophagectomy was performed with a two-field lymphadenectomy. The specimen included the lower and middle mediastinal, subcarinal, and right-sided paratracheal lymph nodes (dissected en bloc). In the abdominal phase, a lymph node dissection identical to the transhiatal approach was performed, as was the construction of a gastric tube and the cervical anastomosis. Finally, a feeding jejunostomy was placed.

### Postoperative Complications

All complications were graded using the modified Clavien-Dindo classification (MCDC) of surgical complications.^16^ Anastomotic leakage included all clinical and radiologic findings of anastomotic dehiscence or fistula. Thoracic empyema and mediastinitis after anastomotic leakage were defined as intrathoracic manifestations of anastomotic leakage.

### Pathologic Analysis

The resected specimen was evaluated using a standard protocol, with emphasis on proximal, distal, and circumferential resection margins, tumor type, extension of the tumor, and the presence and localization of lymph nodes. The 7th edition of the International Union Against Cancer (UICC) was used for tumor-node-metastasis (TNM) classification, tumor grade, and stage grouping.^17^

### Recurrent Disease

For patients with recurrent esophageal cancer, the same protocol was used in all centers. First-line palliative chemotherapy treatment consisted of capecitabine and oxaliplatin chemotherapy. Second-line chemotherapy consisted of paclitaxel with ramucirumab or rinotecan or docetaxel. Irradiation was used to relieve symptoms of metastases.

### Statistical Analysis

Statistical analysis was performed using SPSS version 23.0 (SPSS, Chicago, IL, USA) To evaluate the significance of differences between the two groups, the χ^2^ test was used for categorical variables, and the Mann–Whitney *U* test was used for nonparametric continuous variables. Disease-free and overall survival were analyzed using Kaplan–Meier curves. Differences in survival were analyzed using the log-rank test. A *p* value lower than 0.05 was considered statistically significant.

## Results

### Patient Characteristics

Between April 2005 and November 2011, 176 patients underwent nCRT, and 137 patients underwent pCT followed by esophagectomy. Baseline characteristics did not differ significantly (Table 1). The baseline characteristics were representative for patients with esophageal or junctional adenocarcinoma in West European countries. Supplemental Fig. 1 shows a flow chart in which the clinical course of patients from both groups is shown.

**Table 1:** Baseline characteristics (*n* = 313)

	Chemoradiotherapy (*n* = 176) *n*(%)	Chemotherapy (*n* = 137) *n*(%)	*p* value
Age (years)	63	63	0.570
Gender			
Male	147 (83.5)	113 (82.5)	0.808
Female	29 (16.5)	24 (17.5)	
BMI (kg/m^2^)	25.9	26.2	0.175
Comorbidity			
Vascular	79 (44.8)	63 (46.0)	0.846
Cardiac	36 (20.4)	31 (22.6)	0.642
Pulmonal	17 (9.7)	17 (12.4)	0.438
Oncologic	12 (6.8)	10 (7.3)	0.869
ASA score			
1	35 (19.9)	27 (19.7)	0.781
2	112 (63.6)	91 (66.4)	
3	28 (15.9)	19 (13.9)	
4	1 (0.6)	0 (0)	
Histology			
Adenocarcinoma	176 (100)	137 (100)	
Location tumor			
Mid/distal esophagus	129 (73.3)	104 (75.9)	0.589
GEJ	47 (26.7)	33 (24.1)	
Neoadjuvant treatment			
Chemoradiotherapy (CROSS)	176 (100)		
Chemotherapy (MAGIC)		137 (100)	
ECX		116 (84,7)	
EOX		19 (13.9)	
ECF		2 (1,5)	
Clinical stage			
1	13 (7.4)	5 (3.6)	0.216
2	47 (26.7)	31 (22.6)	
3	116 (65.9)	101 (73.7)	
Surgical approach			
No operation	4 (2.3)	6 (4.4)	0.270
Transthoracic	115 (65.3)	78 (56.9)	
Transhiatal	57 (32.4)	52 (38.0)	
Total gastrectomy with distal esophagectomy	0 (0.0)	1 (0.7)	

*BMI* body mass index, *ASA* American Society of Anesthesiology, *GEJ* gastroesophageal junction, *ECX* epirubicin, cisplatin, and capecitabine, *EOX* epirubicin, oxaliplatin, and capecitabine, *ECF* epirubicin, cisplatin, and fluorouracil

### Toxicity Profile

As a first determinant of toxicity, the ability to complete the delivery of the planned treatment schedule was assessed (Supplemental Fig. 2). The full five cycles of nCRT were administered to 162 (92%) of 176 patients. Of 137 patients, 105 (76.6%) received the full treatment regimen of three preoperative cycles of chemotherapy (*p* = 0.000) (Supplemental Fig. 2). Postoperative continuation of chemotherapy was started for 60 patients (43.8%). The proportion of patients who underwent surgery after initiation of neoadjuvant therapy with curative intent was comparable in the two groups (97.7% after nCRT vs. 95.6% after pCT; *p* = 0.293).

Whereas nCRT was associated with a higher rate of grades 3 and 4 esophagitis (*p* = 0.000), pCT was associated with a higher rate of grades 3 and 4 thromboembolic events (*p* = 0.000), febrile neutropenia (*p* = 0.038), nausea (*p* = 0.001), vomiting (*p* = 0.001), diarrhea (*p* = 0.001), hand–foot syndrome (*p* = 0.005), mucositis (*p* = 0.005), cardiac complications (*p* = 0.002), and electrolyte imbalances. Two patients in the pCT group died during neoadjuvant treatment due to febrile neutropenia (grade 5 toxicity). Pre- and postoperative toxicity for the patients who underwent pCT are shown in Table 2.

**Table 2:** Hematologic toxicity and nonhematologic toxicity (grades 3, 4, and 5)

Preoperative toxicity						Postoperative toxicity	
Cohort	Chemoradiotherapy (*n* = 176)		Chemotherapy (*n* = 137)		*p* Value	Chemotherapy (*n* = 60)	
	Grades 3 and 4 *n* (%)	Grade 5 *n * (%)	Grades 3 and 4	Grade 5		Grades 3 and 4 *n* (%)	Grade 5
Thromboembolic event	1 (0.6)		22 (16.1)		0.000	2 (3.3)	
Neutropenia	10 (5.7)		15 (10.9)		0.088	5 (8.5)	
Leukopenia	20 (11.4)		14 (10.2)		0.747	3 (5.0)	
Nausea	2 (1.1)		13 (9.5)		0.001	8 (13.3)	
Vomiting	2 (1.1)		13 (9.5)		0.001	8 (13.3)	
Diarrhea	0 (0.0)		9 (6.6)		0.001	2 (3.3)	
Febrile neutropenia	0 (0.0)		3 (2.2)	2 (1.5)	0.038	1 (1.7)	
Hand–foot syndrome	0 (0.0)		6 (4.4)		0.005	2 (3.3)	
Mucositis	0 (0.0)		6 (4.4)		0.005	3 (5.0)	
Dehydration	2 (1.1)		4 (2.9)		0.254	1 (1.7)	
Cardiac complications	0 (0.0)		7 (5.1)		0.002	1 (1,7)	
Hyponatremia	0 (0.0)		6 (4.4)		0.005	0 (0.0)	
Hypokalemia	0 (0.0)		4 (2.9)		0.023	0 (0.0)	
Anemia	1 (0.6)		2 (1.5)		0.422	0 (0.0)	
Thrombocytopenia	4 (2.3)		1 (0.7)		0.280	0 (0.0)	
Urinary tract infection	0 (0.0)		1 (0.7)		0.256	0 (0.0)	
Allergic reaction	0 (0.0)		1 (0.7)		0.256	0 (0.0)	
Anorexia	2 (1.1)		1 (0.7)		0.714	0 (0.0)	
Respiratory infection	0 (0.0)		1 (0.7)		0.256	1 (1,7)	
Peripheral neuropathy	0 (0.0)		2 (1.5)		0.108	2 (3.3)	
Fatigue	3 (1.7)		0 (0.0)		0.125	3 (5.0)	
Esophagitis	19 (11.0)		0 (0.0)		0.000	0 (0.0)	
Hypophosphatemia	0 (0.0)		1 (0.7)		0.256	0 (0.0)	
Tinnitus	0 (0.0)		0 (0.0)		1.000	1 (1,7)	

### Postoperative Complications

The surgical results and postoperative complications are shown in Table 3. In the chemoradiotherapy group, 104 (60.5%) of 172 patients had a complicated course, whereas in the chemotherapy group, a complicated postoperative course was observed in 79 (60.3%) of 131 patients (*p* = 0.978). The incidence of postoperative cardiac complications was significantly higher in the chemoradiotherapy group than in the chemotherapy group (17.4% vs. 6.9%; *p* = 0.006). The incidences of all other postoperative complications were comparable between the two groups. There were no differences in median overall Clavien-Dindo complication grades. The postoperative hospital stay was 11 days in the nCRT group and 13 days in the PCT group (*p* = 0.224). Postoperative overall in-hospital mortality did not differ significantly between the chemoradiotherapy and chemotherapy groups (4.7% vs. 2.3%, respectively; *p* = 0.276) (Table 3).

**Table 3:** Postoperative complications (*n* = 303)

	Chemoradiotherapy (*n* = 172) *n* (%)	Chemotherapy (*n* = 131) *n* (%)	*p* value
Complications	104 (60.5)	79 (60.3)	0.978
No complications	68 (39.5)	52 (39.7)	
Pneumonia	35 (20.3)	39 (29.8)	0.059
Pulmonary embolism	2 (1.2)	6 (4.6)	0.066
Anastomotic leakage	22 (12.8)	25 (19.1)	0.134
Cardiac complications	30 (17.4)	9 (6.9)	0.006
Chylothorax	14 (8.1)	16 (12.2)	0.252
Vocal cord paralysis	21 (12.2)	11 (8.4)	0.285
Bleeding	1 (0.6)	4 (3.1)	0.094
Wound infection	3 (1.7)	7 (5.3)	0.082
In-hospital mortality	8 (4.7)	3 (2.3)	0.276

### Pathologic Results

The pathologic results are shown in Table 4. A R0 resection was achieved for 160 (93%) of 172 patients in the chemoradiotherapy group, compared with 120 (91.6%) of 131 patients in the chemotherapy group (*p* = 0.644). In the chemoradiotherapy group, significantly more downstaging occurred, with lower ypT-stages and more favorable tumor regression grades than in the chemotherapy group (*p* = 0.007 and 0.000, respectively) (Table 4).

**Table 4:** Surgical and pathologic results (*n* = 303)

	Chemoradiotherapy (*n* = 172) *n* (%)	Chemotherapy (*n* = 131) *n* (%)	*p* Value
Response			
Complete response (Mandard 1)	26 (15.1)	9 (6.9)	0.000
Partial response (Mandard 2,3)	99 (57.6)	38 (29.0)	
No response (Mandard 4,5)	47 (27.3)	85 (64.1)	
Radicality			
R0	160 (93.0)	120 (91.6)	0.644
R1	12 (7.0)	11 (8.4)	
Lymph nodes			
Median: *n* (range)	20	22	0.738
Pathologic stage			
0	26 (15.1)	9 (6.9)	0.007
1	34 (19.8)	16 (12.2)	
2	50 (29.1)	36 (27.5)	
3	62 (36.0)	70 (53.4)	

### Survival and Recurrence

**Fig 1:**
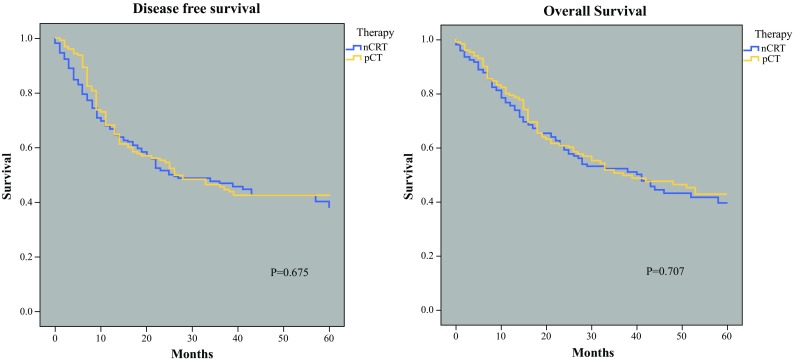
Disease free and overall survival for neoadjuvant chemoradiotherapy (nCRT) and perioperative chemotherapy (pCT). Median follow up was 42 months for nCRT and 41 months for pCT

All the patients were included in the survival analysis (Fig. 1). The median follow-up time was 42 months for nCRT and 41 months for pCT. The median overall survival was 41 months after nCRT versus 37 months after pCT (*p* = 0.707). The median disease-free survival time was 26 months for both the nCRT and pCT groups (*p* = 0.675).

In the nCRT group, 98 (57%) of 172 patients had no signs of recurrent disease. In the pCT group 64 (49%) of 131 patients did not have recurrent disease (*p* = 0.467). Locoregional recurrence was observed in 8 patients (5%) in the nCRT group versus 10 patients (8%) in the pCT group. Distant metastases were observed in 45 patients (26%) in the nCRT group and 38 patients (19%) in the pCT group. Combined locoregional and distant metastases were observed in 21 patients (12%) in the nCRT group versus 19 (15%) patients in the pCT group (Supplemental Table 3).

## Discussion

This is the first study to compare neoadjuvant treatment with chemoradiotherapy using paclitaxel, carboplatin plus concurrent radiotherapy (CROSS^8^), and perioperative chemotherapy consisting of epirubicin, cisplatin, and capecitabin (MAGIC^4^) for patients with resectable adenocarcinoma of the esophagus or GEJ. Although nCRT was associated with better tumor downstaging and more favorable tumor regression grades, equal rates of radical resections and comparable disease-free and overall survival outcomes were observed. The risk of serious adverse events and the necessity to interrupt treatment were significantly higher for the patients treated with pCT.

### Oncologic Results and Survival

Both perioperative chemotherapy and neoadjuvant chemoradiotherapy have been found to improve survival for adenocarcinoma compared with surgery alone.^4^–^6^^,^ 8 ^,^ 9 In a meta-analysis by Sjoquist et al.^9^, two small randomized controlled trials directly comparing neoadjuvant chemoradiotherapy with chemotherapy were included. These two trials showed similar R0 resection rates between treatment groups but significantly higher pathologically complete response rates and lower locoregional recurrence rates in the neoadjuvant chemoradiotherapy plus surgery groups. However, these findings did not result in a survival benefit for nCRT compared with pCT.^10^^,^ 11

Our study showed similar results. We demonstrated that nCRT leads to better downstaging of esophageal adenocarcinomas than pCT, without differences in R0 resection rates between nCRT (93%) and pCT (92%) (*p* = 0.644). No statistically significant differences in the risk of locoregional tumor recurrence, disease-free survival, or overall survival between nCRT and pCT at the long term follow-up assessment were found. After a follow-up period of approximately 41 months, the median disease-free survival period was 26 months for both nCRT and pCT (*p* = 0.675). The median overall survival period was 41 months after nCRT versus 37 months after pCT (*p* = 0.707). Interestingly, the survival results for pCT used to treat esophageal adenocarcinoma were better than reported earlier, which could be attributed to improved preoperative staging techniques, improved perioperative care, and centralization of esophageal surgery.^4^^,^ 18

Based on the results of this study and in accordance with the literature, we conclude that nCRT and pCT are equally safe in terms of oncologic outcomes.

### Toxicity and Postoperative Complications

As a first determinant of toxicity, we assessed the ability to deliver the planned treatment schedule. Whereas 92% of the nCRT patients completed the planned protocol, only 31.8% of the patients completed the pCT schedule, emphasizing the low feasibility of the postoperative chemotherapy courses for patients with esophageal and GEJ adenocarcinoma. This can be attributed to the initial performance status and the morbidity associated with esophageal surgery and the higher level of toxicity associated with pCT.^14^ Compared with nCRT, in our series, pCT led to a wider range and a higher frequency of severe adverse events.

Another important clinical parameter was the incidence of postoperative complications after nCRT or pCT. Except for a higher incidence of cardiac complications in the nCRT group, no statistically significant differences in postoperative complications or postoperative mortalitiy were observed between nCRT and pCT. This corresponds with the results from a meta-analysis describing postoperative morbidity and perioperative mortality in patients receiving neoadjuvant chemotherapy or chemoradiotherapy for resectable esophageal and GEJ cancers.^19^

This observation was confirmed in a recent study by Klevebro et al.^12^ in which 181 patients with esophageal adenocarcinoma and squamous cell carcinoma were randomized to neoadjuvant chemotherapy or neoadjuvant chemoradiotherapy. These authors concluded that neoadjuvant chemoradiotherapy was not associated with a higher overall incidence of postoperative complications or postoperative mortality after esophagectomy compared with chemotherapy. However the complications that occurred for the patients who received chemoradiotherapy were more severe, with a higher median Clavien-Dindo score. This provides a level of ambiguity regarding the safety of adding radiotherapy as an adjunct to the neoadjuvant chemotherapy.

With no difference in oncologic outcomes, it could be argued what the indication might be for both therapies. The rationale for the addition of irradiation to chemotherapy for resectable esophageal carcinoma is based on good evidence of increased tumor downsizing and improved local control.^11^

Besides local control and downstaging, it is remarkable that carboplatin- and paclitaxel-based nCRT also exhibits a profound systemic effect, which is reflected by a comparable percentage of systemic metastases, as shown after pCT. This finding is supported by recent studies in which the systemic effect of these agents was demonstrated in both locoregional and metastatic adenocarcinoma of the esophagus.^20^^,^ 21

Well-powered randomized controlled trials with long-term follow-up evaluation are needed to address the question of which therapy regimen is preferable for the treatment of resectable esophageal adenocarcinoma. The Neo-AEGIS randomized clinical trial (NCT01726452) directly compares the nCRT (CROSS) regimen with the pCT (MAGIC) regimen as described in our studies. The results are awaited in the coming years.^22^

Finally, comparing results between high-volume referral centers for esophageal carcinoma might have introduced bias by indication, surgery, and postoperative care differences. However, the process of data validation by two separate authors and comparable surgical training might have limited the amount of bias in this study. Furthermore, our data represented consecutive patients in all centers, so the role of patient selection was minimized.

In conclusion, for patients with esophageal or GEJ adenocarcinoma, chemoradiotherapy with paclitaxel, carboplatin, and concurrent radiotherapy and perioperative chemotherapy with epirubicin, cisplatin, and capecitabin lead to equal oncologic outcomes in terms of radical resection rates, lymphadenectomy, patterns of recurrent disease, and (disease-free) survival. However, neoadjuvant chemoradiotherapy is associated with a considerably lower level of severe adverse events and should therefore be the preferred protocol until a well-powered randomized controlled trial provides different insights.

## Electronic Supplementary Material

Below is the link to the electronic supplementary material.

Supplementary material 1 (JPEG 41 kb)

Supplementary material 2 (JPEG 74 kb)

Supplementary material 3 (DOC 27 kb)
