# Supplementary figures and images for: Preoperative Chemoradiotherapy Versus Perioperative Chemotherapy for Patients With Resectable Esophageal or Gastroesophageal Junction Adenocarcinoma
Source: Ann Surg Oncol. 2017 Apr 19;24(8):2282–90. doi: 10.1245/s10434-017-5827-1 (PMC5491642; doi:10.1245/s10434-017-5827-1)

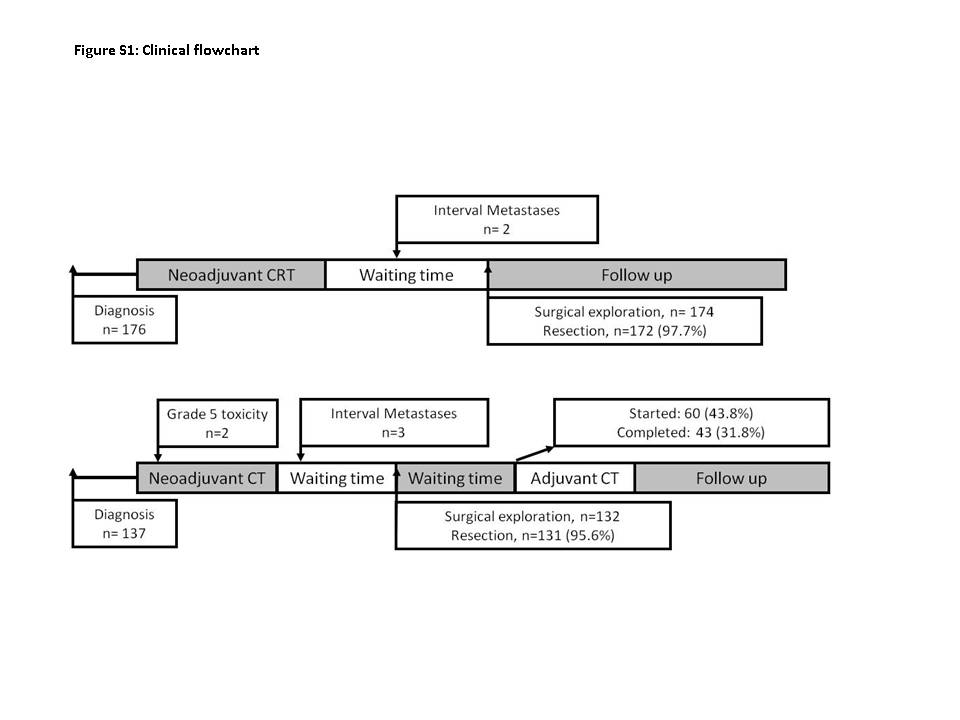

Supplement: Supplementary file 1 — Supplementary material 1 (JPEG 41 kb) [file 10434_2017_5827_MOESM1_ESM.jpg]

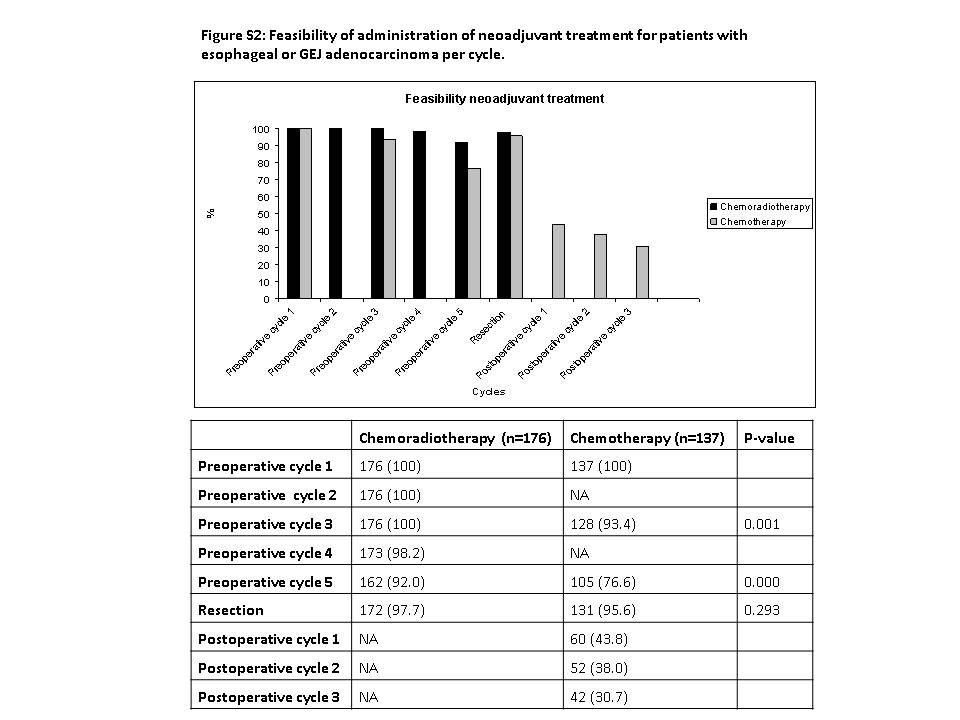

Supplement: Supplementary file 2 — Supplementary material 2 (JPEG 74 kb) [file 10434_2017_5827_MOESM2_ESM.jpg]
