# Supplementary material for: Preoperative Chemoradiotherapy Versus Perioperative Chemotherapy for Patients With Resectable Esophageal or Gastroesophageal Junction Adenocarcinoma
Source: Ann Surg Oncol. 2017 Apr 19;24(8):2282–90. doi: 10.1245/s10434-017-5827-1 (PMC5491642; doi:10.1245/s10434-017-5827-1)
Supplement: Supplementary file 3 — Supplementary material 3 (DOC 27 kb) [file 10434_2017_5827_MOESM3_ESM.doc]

| **Table S1. Disease recurrence (n=303) (Supplemental 2, S2)** | |  | |
| --- | --- | --- | --- |
|  | **Chemoradiotherapy (n=172)** | **Chemotherapy (n =131)** | **P-value** |
| **No Recurrence** | 98 (57) | 64 (49) | 0.467 |
| **Recurrence** |  |  |  |
| Locoregional | 8 (5) | 10 (8) |  |
| Distant | 45 (26) | 38 (29) |  |
| Both | 21 (12) | 19 (15) |  |
